# Is the Perinatal Outcome of Placental Abruption Modified by Clinical Presentation?

**DOI:** 10.1155/2011/659615

**Published:** 2010-10-05

**Authors:** Seishi Furukawa, Hiroshi Sameshima, Tsuyomu Ikenoue, Masanao Ohashi, Yoshio Nagai

**Affiliations:** ^1^Department of Obstetric & Gynecology, Faculty of Medicine, University of Miyazaki, Miyazaki 889-1692, Japan; ^2^Department of Obstetric & Gynecology, Miyazaki Medical Association Hospital, Miyazaki 889-1692, Japan

## Abstract

*Objective*. The purpose of this study was to elucidate the impact of the clinical presentation on perinatal outcome in placental abruption. *Study Design*. A retrospective study was performed in 97 placental abruptions. Placental abruptions were classified according to clinical presentation: pregnancy-induced hypertension (HT, *n* = 22), threatened premature labor and/or premature rupture of membranes (TPL/ROM, *n* = 35), clinically low risk (LR, *n* = 27), and others (*n* = 13). Perinatal outcomes were compared among the HT, TPL/ROM, and LR groups. *Results*. The HT had significantly higher incidence of IUGR, IFUD, and low fibrinogen. The TPL/ROM had less severe disease. However, the LR had significantly higher incidence of IUFD, low UA pH < 7.1, low Apgar score of <7 at 5 min, and low fibrinogen. *Conclusion*. Disease severity in placental abruption is likely to depend on the clinical presentation.

## 1. Introduction

Placental abruption is a major cause of poor perinatal outcome [1–3] and occurs in approximately 1% of all pregnancies [4, 5]. In order to elucidate the etiology of placental abruption, maternal risk factors and episodes of placental abruption need to be evaluated. To date, at least 2 major pathways have been proposed to account for placental abruption: (1) inflammation-associated processes and (2) ischemia-associated process [6]. For example, women presenting with preterm premature rupture of membranes (preterm PROM) are at increased risk of developing abruption, and histologic chorioamnionitis is associated with placental abruption [7, 8]. Poor trophoblast invasion to decidua accounts for the etiology of placental abruption [9, 10], and it is now agreed that placental abruption occurs with increasing frequency in patients with chronic hypertension [11]. Thus, preterm PROM, threatened premature labor, and hypertension are major risk factors correlated with placental abruption, although placental abruption also occurs in women without the known risk factors. Although the frequency of placental abruption is higher in women with preterm PROM or pregnancy-induced hypertension, it is unknown if the severity of placental abruption increases in such cases.

Attempts have been made to correlate the perinatal outcome of placental abruption with single risk factors such as prematurity, hypertension, inflammation, and multiple pregnancy [12–15]. In these studies, coexisting risk factors that may contribute to the development of placental abruption were not excluded and therefore may have led to a misinterpretation of the results. Furthermore, only a few reports have compared perinatal outcome in placental abruption among groups having different clinical presentations such as genital bleeding and female gender of the fetus [16, 17]. Therefore, we compared the perinatal outcome of placental abruption among different clinical presentations.

## 2. Materials and Methods

We retrospectively reviewed medical charts of women with placental abruption that were admitted to the Perinatal Center of the University of Miyazaki and Miyazaki Medical Association Hospital from January 1997 to April 2009. University of Miyazaki is a tertiary-level center and Miyazaki Medical Association Hospital is a secondary-level hospital. In our area, 80% of pregnant women give birth at a private clinic. Both centers mainly dealt with referral cases from private clinics, and the total number of deliveries was 8557. In this study, we determined placental abruption by the presence of retroplacental hematoma and clinical presentations (any one or combination of genital bleeding, abdominal pain, pregnancy-induced hypertension, premature labor, premature rupture of membrane, intrauterine fetal death, or non-reassuring fetal heart rate pattern). Confirmation of retroplacental hematoma was made by visual examination after delivery. If hematoma was evident immediately after detachment of the placenta, this was also regarded as retroplacental hematoma. If clotting was incidentally founded in the absence of any clinical signs, the case was excluded. Cases involving multiple pregnancies were also excluded. Finally, a total of 97 pregnancies displaying placental abruption were included.

In this study, chronic hypertension was defined as hypertension diagnosed prior to conception or within the first 20 weeks of pregnancy. Pregnancy-induced hypertension such as gestational hypertension, preeclampsia, eclampsia, and superimposed preeclampsia was defined according to the classification of National High Blood Pressure Education Program Working Group Report on High Blood Pressure in Pregnancy [18]. Women with pregnancy-induced hypertension were managed with maternal blood pressure monitoring (3~4 times/day), weekly renal and liver function tests, daily fetal heart rate monitoring (FHR monitoring), daily fetal movement count, weekly biophysical profile scoring (BPS), and weekly fetal biometry using ultrasonography (USG). Pregnancy was terminated in severe cases such as those involving persistent severe hypertension (blood pressure > 160/110 mmHg), oliguria (<500 mL/day), low platelet count (<100,000/mm^3^), HELLP syndrome (hemolysis, elevated liver enzyme and low platelets), pulmonary edema, or eclampsia. The severity of proteinuria alone was not an indication to terminate pregnancy [19]. Pregnancies were also terminated if there was any evidence of apparent intrauterine growth restriction (IUGR) or non-reassuring fetal heart rate patterns (NRFHR).

Threatened premature labor (TPL) was defined when the patient had uterine contractions of four in 20 minutes or eight in 60 minutes with progressive changes of dilatation and/or effacement in the cervix. Tocolysis was first conducted with continuous intravenous administration of ritodrine hydrochloride. The FHR monitoring was also conducted to detect potential fetal hypoxia prior to the start of tocolysis. Tocolysis was stopped if fetal asphyxia was evident. If uterine contractions continued, magnesium sulfate was added.

We defined NRFHR and low BPS (≤4) as fetal asphyxia. Non-reassuring FHR patterns to terminate pregnancy included recurrent late decelerations, recurrent severe variable decelerations, or prolonged deceleration.

Ninety-seven placental abruptions were then classified into 4 subgroups according to the clinical presentation. Women with pregnancy-induced hypertension who had chronic hypertension were categorized as HT (*n* = 22). Women with threatened premature labor and/or premature rupture of membrane (ROM) were categorized as TPL/ROM (*n* = 35). Women that had other medical or obstetrical risk factors were categorized as others (*n* = 13). Women without the aforementioned risk factors were categorized as clinically low risk (LR, *n* = 27). Women with HT and ROM were regarded as HT. Women admitted to hospital with genital bleeding, abdominal pain, or loss of fetal movement were distinguished from TPL if NRFHR was also diagnosed at admission. Perinatal outcomes were compared among the HT, TPL/ROM, and LR groups. Since others (*n* = 13) had heterogeneous risk factors (Table 1), we omitted them from this study. Thus, this study focused on assigning inflammation-associated processes, ischemia-associated processes, and low risk.

The following clinical characteristics were collected: maternal age, primipara, gestational age at delivery (weeks), birth weight (g), presence of genital bleeding for first awareness, and medical complications such as diabetes mellitus and thyroid disease. Perinatal outcomes including the incidence of fibrinogen at <150 mg/dl, platelet count <10 × 10^4^/mm^3^, cesarean delivery, intrauterine fetal death (IUFD), IUGR, umbilical artery pH < 7.1 (UA pH < 7.1), and an Apgar score of <7 at 5 minutes were also evaluated. The fibrinogen and platelet count values of the mother were obtained at the time of diagnosis of placental abruption. IUGR was defined as sex-specific birth weight less than the 10th percentile for gestational age according to the Japanese standard growth curve for singleton.

We also obtained the pathological findings of placentas for clinical classification. The following findings were obtained for each placenta: chorioamnionitis, ischemic changes, and infarcts. Ischemic changes included chorangiosis, increased syncytial knots, and atherosis of uteroplacental vessels. Necrosis of the decidual tissue and angioectasia, and fibrinoid degeneration of the villi were excluded from consideration as ischemic changes.

Comparisons among groups were made using the one-way ANOVA or *χ* ^2^ tests. Post hoc analysis was performed using the Bonferroni-Dunn test.

## 3. Results

As shown in Table 2, the incidence of primipara was not significant. TPL/ROM was marked by early gestational age compared to HT and LR (*P* < .01 and *P* < .01). However, there was no significant difference in birth weight among the groups. The presence of genital bleeding for first awareness was higher in the TPL/ROM group (*P* = .03  versus HT); however, no difference was observed between the HT and LR groups (*P* = .35).

There was a significant difference in the incidence of IUGR, IUFD, UA pH < 7.1, and fibrinogen at <150 mg/dl (Table 3). The HT group had significantly higher incidence of IUGR, IFUD, and low fibrinogen. The TPL/ROM group did not have any particular trends in risk factors. However, LR group had significantly higher incidence of IUFD, low UA pH < 7.1, low Apgar score of <7 at 5 minutes, and low fibrinogen.

According to the histological findings, chorioamnionitis was most marked in the TPL/ROM group compared to HT and LR (*P* = .02 and *P* = .03, Table 4). The incidence of ischemic changes in the HT group was higher compared to the LR group (*P* = .03). The incidence of infarcts in LR was higher compared to TPL/ROM (*P* < .01).

## 4. Discussion

In this study, we demonstrated a significant difference in perinatal outcome among the HT, TPL/ROM, and LR groups. Surprisingly, clinically low risk women were most likely to have more severe manifestation including a high incidence of IFUD, fibrinogen at <150 mg/dl, UA pH <7.1, and a low Apgar score. The HT group also had a high incidence of IUFD and fibrinogen at <150 mg/dl, although the incidence of low pH and a low Apgar score was lower than that of LR. The TPL/ROM group was marked by the lowest incidence of severe disease among the 3 groups. We showed here the relationship between clinical presentation of women and perinatal outcome with respect to placental abruption.

We divided a group of women with placental abruption into four groups, that is, HT, TPL/ROM, clinically low risk, and others, according to the clinical presentations. We determined the clinical classification based on the etiology of placental abruption. To date, at least 2 major pathways have been proposed to account for placental abruption, inflammation- and ischemia-associated processes [6]. It has been reported that poor trophoblast invasion to decidua accounts for the etiology of placental abruption [9, 10], and the pathological findings of placentas are common to both placental abruption and pregnancy-induced hypertension [11]. Thus, we regarded pregnancy-induced hypertension and chronic hypertension as belonging to the ischemic process. In terms of inflammatory processes, it has been reported that women presenting with preterm PROM are at increased risk of developing abruption [7]. Nath et al. reported that histologic chorioamnionitis is associated with placental abruption [8]. Thus, we regarded premature rupture of the membrane and premature labor as belonging to inflammation-associated processes. Since the risk factors were not uniform in other group, this was omitted from this study. This study focused on assigning inflammation- and ischemia-associated processes. To validate our classification, placentas were checked for pathological lesions. Consistent with the histological records, chorioamnionitis was most noted in the TPL/ROM group compared to the HT and LR groups. In addition, the incidence of ischemic changes in the HT group was higher compared to the LR group. As a result, our clinical classification was shown to be consistent with the histological examinations.

We previously reported perinatal outcomes in low risk pregnancies over 32 weeks' of gestation [20]. We found 9 children with cerebral palsy (CP) out of 5522 low risk pregnancies. Among these, two were cytomegalovirus infection, one was amniotic fluid embolism during labor, and the other 6 were placental abruption. These 6 women with placental abruption showed non-reassuring fetal heart rate patterns on admission. On the other hand, 81 women developed placental abruption after hospitalization, and there were no cases of children who developed CP. Since women with high risk factors such as hypertension, endocrine disorders or multiple pregnancies were excluded, 81 women were hospitalized due only to TPL. Our study also showed the TPL/ROM group was marked by the lowest incidence of severe disease. On the other hand, the HT and clinically low risk groups were marked by severe disease. Thus, perinatal outcome of placental abruption is associated with clinical presentation of the mother during pregnancy.

In terms of perinatal outcome of placental abruption, Morgan et al. showed that no overall difference in perinatal outcome was observed between groups with or without hypertension [13]. This is partially consistent with our results. In our study, although maternal morbidity in HT was similar to the low risk group, the low risk women experiencing placental abruption were more likely to have low UA pH and low Apgar scores. This difference is partially due to nonhospitalization, as the low risk women were not hospitalized, leading to delayed care. We also found that clinically low risk women showed higher incidence of fibrinogen consumption. In addition, clinically low risk women more likely have “concealed hemorrhage”, since the presence of genital bleeding for first awareness was fewer in the HT and LR groups compared to the TPL/ROM group. This is not only due to consumptive coagulopathy, but also due to the fact that the extent of hemorrhage is not readily discovered and the diagnosis is delayed [21]. However, an explanation as to why clinically low risk women without ischemia or inflammation were likely to have severe disease could not be clearly ascertained in this study. Unknown factors independent of ischemia and inflammation may operate in the development of placental abruption. Interestingly, we observed that clinically low risk women had infarcts in placentas without ischemic changes although infarcts were reported less prominent in women without hypertension [22]. Thus, a new pathway leading to placental abruption may be associated with the etiology of placental infarction. Further studies are required to elucidate the mechanisms leading to placental abruption in the absence of ischemia or inflammation.

In conclusion, we showed the relationship between some clinical presentations and poor outcome in women with placental abruption. Clinically low risk women have similar risk to have poor outcome compared to the HT group. The TPL/ROM group had the least severe disease among the 3 groups. In a clinical situation, opportunities exist for early recognition and prompt deliveries in cases where the mother has a clinical presentation of hypertension, TPL, or ROM.

##  Conflict of Interests

There is no financial or other relationship that might lead to a conflict of interests.

## Figures and Tables

**Table 1:** Medical and obstetrical complications in the others group.

Number	13	Perinatal outcome		
		IUFD	UA pH < 7.1 among live births	Fibrinogen < 150 mg/dl
*Medical complications*				
Asthma	1		1	1
Chronic nephritis	1			
Smoker (>1 pack per day)	2	1	1	2
*Obstetrical complications*				
Prior cesarean section	3	1	1	1
Prior placental abruption	2 (1: prior cesarean section)		2	1
IUGR without HT and oligohydramnios	1			
Oligohydramnios without HT and ROM	3		2	

IUGR: intrauterine growth restriction, HT: pregnancy induced hypertension, and ROM: premature rupture of membrane.

**Table 2:** Demographic data of women complicated with placental abruption among the HT, TPL/ROM, and clinically low risk groups.

	HT	TPL/ROM	Clinically low risk
Number	22	35	27
Age (yr)	31.4 ± 6.5^†^	27.9 ± 5.2	28.4 ± 4.1
Nulliparity (%)	13 (59%)	19 (54%)	11 (41%)
Gestational age at delivery (wk)	34.2 ± 4.0^†^	31.3 ± 4.4	34.4 ± 3.4^†^
Birth weight (g)	2097 ± 747	1767 ± 704	2200 ± 548
Genital bleeding for the first symptom	2*	12	5
Medical complication			
DM	1	0	0
Thyroid disease	3	0	0

Results are expressed as Mean ± SD or incidence (%). HT: pregnancy-induced hypertension, TPL/ROM: threatened premature labor and/or premature rupture of membrane, and DM: diabetes mellitus.

*P < .05 versus TPL/ROM by *χ* ^2^ test. ^†^ *P* < .05 versus TPL/ROM by Bonferroni/Dunn analysis.

**Table 3:** The perinatal outcome of women complicated with placental abruption among groups of HT, TPL/ROM, and clinically low risk.

	HT	TPL/ROM	Clinically low risk
Number	22	35	27
Cesarean delivery (%)	16 (73%)	27 (77%)	23 (85%)
IUGR (%)	10 (45%)*	2 (6%)	4 (15%)
IUFD (%)	6 (27%)*	0 (0%)	7 (26%)*
UA pH < 7.1 among live births (%)	3 (19%)	2 (6%)	11 (55%)^∗†^
Low Apgar score at 5 min (less than 7) among live births (%)	1 (6%)	4 (11%)	10 (50%)^∗†^
Fibrinogen < 150 mg/dl	6 (27%)*	0 (0%)	10 (37%)*
Platelet count < 10 × 10^4^/mm^3^	3 (14%)	1 (3%)	1 (4%)

Results are expressed as Mean ± SD or incidence (%). HT: pregnancy-induced hypertension and TPL/ROM: threatened premature labor and/or premature rupture of membrane.

*P < .05 versus TPL/ROM by *χ* ^2^ test. ^†^ *P* < .05 versus HT by *χ* ^2^ test.

**Table 4:** The histological study of women complicated with placental abruption among groups of HT, TPL/ROM, and clinically low risk.

	HT	TPL/ROM	Clinically low risk
Number	22	35	27
(No histological records)	(6)	(4)	(3)
Chorioamnionitis (%)	1 (6%)*	12 (39%)	3 (13%)*
Ischemic changes (%)	7 (44%)	7 (23%)	3 (13%)^†^
Infarcts (%)	5 (31%)	3 (10%)	10 (42%)*

Results are expressed as Mean ± SD or incidence (%). HT: pregnancy-induced hypertension and TPL/ROM: threatened premature labor and/or premature rupture of membrane.

*P < .05 versus TPL/ROM by *χ* ^2^ test. ^†^ *P* < .05 versus HT by *χ* ^2^ test.
